# *Mytilus trossulus* introgression and consequences for shell traits in longline cultivated mussels

**DOI:** 10.1111/eva.13245

**Published:** 2021-05-10

**Authors:** Kati Michalek, David L. J. Vendrami, Michaël Bekaert, David H. Green, Kim S. Last, Luca Telesca, Thomas A. Wilding, Joseph I. Hoffman

**Affiliations:** ^1^ The Scottish Association for Marine Science Oban UK; ^2^ Department of Animal Behaviour University of Bielefeld Bielefeld Germany; ^3^ Institute of Aquaculture Faculty of Natural Sciences University of Stirling Stirling UK; ^4^ Department of Earth Sciences University of Cambridge Cambridge UK; ^5^ British Antarctic Survey Cambridge UK; ^6^ Present address: Lamont‐Doherty Earth Observatory of Columbia University Palisades NY USA

**Keywords:** geometric morphometrics, introgressive hybridization, Me15/16, *Mytilus edulis* species complex, shellfish aquaculture, single nucleotide polymorphism

## Abstract

Mussels belonging to the *Mytilus* species complex (*M*. *edulis*, *ME*; *M*. *galloprovincialis*, *MG*; and *M*. *trossulus*, *MT*) often occur in sympatry, facilitating introgressive hybridization. This may be further promoted by mussel aquaculture practices, with *MT* introgression often resulting in commercially unfavourable traits such as low meat yield and weak shells. To investigate the relationship between genotype and shell phenotype, genetic and morphological variability was quantified across depth (1 m to 7 m) along a cultivation rope at a mussel farm on the West coast of Scotland. A single nuclear marker (Me15/16) and a novel panel of 33 *MT*‐diagnostic single nucleotide polymorphisms were used to evaluate stock structure and the extent of *MT* introgression across depth. Variation in shell strength, determined as the maximum compression force for shell puncture, and shell shape using geometric morphometric analysis were evaluated in relation to cultivation depth and the genetic profiles of the mussels. Overall, *ME* was the dominant genotype across depth, followed by *ME* × *MG* hybrids and smaller quantities of *ME* × *MT* hybrids and pure *MT* individuals. In parallel, we identified multiple individuals that were either predominantly homozygous or heterozygous for *MT*‐diagnostic alleles, likely representing pure *MT* and first‐generation *ME* × *MT* hybrids, respectively. Both the proportion of individuals carrying *MT* alleles and *MT* allele frequency declined with depth. Furthermore, *MT*‐introgressed individuals had significantly weaker and more elongate shells than nonintrogressed individuals. This study provides detailed insights into stock structure along a cultivation rope and suggests that practical methods to assess shell strength and shape of cultivated mussels may facilitate the rapid identification of *MT*, limiting the impact of this commercially damaging species.

## 1 INTRODUCTION

In the Northern Hemisphere, there are three sister species of morphologically similar but genetically distinct blue mussels—*Mytilus edulis* (Linnaeus, 1758; hereafter *ME*), *M*. *galloprovincialis* (Lamarck, 1819; *MG*) and *M*. *trossulus* (Gould, 1850; *MT*) which collectively form the *Mytilus edulis* species complex. Their global and local distribution are well understood (Gosling, 2003; McDonald et al., 1991) and are mostly correlated with sea surface temperature over large spatial scales (Fly & Hilbish, 2013) and salinity over local scales (Kijewski et al., 2019; Sarver & Foltz, 1993; Telesca et al., 2018). Importantly, the distributions of these species often overlap, facilitating interbreeding and the formation of hybrid zones in areas of sympatric occurrence (Gosling, 1992; Vendrami et al., 2020). The best known hybrid zone exists between *ME* and *MG*, which occur intermittently along the coastlines from southern France to Scotland (Bierne et al., 2003; Skibinski et al., 1983), whilst hybrids between *ME* and *MT* are more prevalent in cooler waters of the North Pacific, Northwest Atlantic and parts of Northern Europe (Katolikova et al., 2016; Riginos & Cunningham, 2005; Väinölä & Strelkov, 2011; Wenne et al., 2016).

Both *ME* and *MG* are extensively cultivated across Europe, with an annual production of nearly half a million tonnes (Avdelas et al., 2020). Cultivation sites either utilize areas of the seabed (‘bottom culture’) or support mussels in the water column using poles (‘bouchot culture’), longlines and rafts (‘suspended rope culture’). In Scotland, mussels are rope‐grown in fjordic inlets (sea lochs and voes) with restricted exchange with open ocean waters, resulting in a high degree of seasonally modified stratification (Edwards & Sharples, 1986). A typical cultivation system consists of a series of submerged longlines ‘headers’ (100–200 m length) oriented parallel to the shore, from which the growing lines (‘droppers’, 4–8 m length) are strung vertically in regular intervals. Production depends exclusively on the settlement of natural mussel larvae or ‘spat’ onto the growing structures or designated spat collectors. These are later stripped and reseeded onto droppers, and the mussels are left to grow until harvesting 2 to 3 years later (Scott et al., 2010). Suspended rope culture provides large surface areas for mussel settlement, facilitating larval dispersal and gamete mixing, whilst benthic predation is largely excluded. However, spat movements between farm sites, within or outside the same water body, may facilitate gene flow between species and favour introgressive hybridization in farmed and nearby wild mussel populations (Michalek et al., 2016).

In Scotland, interbreeding and introgression between *ME* and *MT* are a particular concern for the shellfish sector, which is a relatively small yet important industry in supporting employment in rural and often remote areas (6699 tonnes valued at £6.2 million in 2019; Munro, 2020). Whilst mixed‐species stocks of *ME* and *MG* are cultivated successfully along the Scottish west coast, several farm sites have experienced an increased occurrence of mussels morphologically different from native stocks, which were subsequently identified as pure *MT* or *ME* × *MT* hybrids, respectively (Beaumont et al., 2008). When compared with *ME* and *MG*, *MT* has often been associated with thin fragile shells, which easily break during harvest and processing, lowered meat yields, reduced shelf life and with more elongated shells, all of which constitute undesired traits for mussel production (Dias, Bland et al., 2009; Mallet & Carver, 1995; Penney et al., 2007). After initial reports of *MT* in Loch Etive, Argyll, in 2004 (Beaumont et al., 2008), *MT* and *ME* × *MT* became the dominant species on on‐growing facilities, leading to significant losses in production (only 28 tonnes were produced in 2009 compared with 240 tonnes in 2002) making mussel farming in the area economically unviable (Gubbins et al., 2012). Indeed, *MT* is now classified as a commercially damaging species in Scotland (‘The Aquaculture and Fisheries (Scotland) Act 2013’) where its presence is notifiable to mitigate future impacts on the industry (Gubbins et al., 2012).

Such management efforts depend on reliable species identification. A variety of genetic markers are available today for genotyping *Mytilus* species, of which the most routinely used is the nuclear DNA marker Me15/16 (Inoue et al., 1995) which amplifies a species‐specific diagnostic region of the adhesive protein gene. Using this marker in surveys of wild and farmed *Mytilus* populations across Scotland, *MG*, *MT* and *MG* × *MT* hybrids were rare (Dias et al., 2008). However, *MT* and *ME* × *MT* hybrids were present and appeared to occur at higher frequencies in sheltered environments, such as marinas and aquaculture structures when compared to exposed intertidal habitats (Dias, Dordor et al., 2009) and were often associated with lower seawater salinities (Beaumont et al., 2008). In contrast to genotyping at a single locus, multi‐locus genotyping of single nucleotide polymorphisms (SNPs) provides greater resolution to determine the proportion of the genome belonging to each genetic background (Morin et al., 2004; Twyford & Ennos, 2012) allowing for the analysis of introgressive hybridization. SNP assays applied in *Mytilus* population studies have revealed differences in species distribution and genotypic composition with location and environmental variation (Vendrami et al., 2020; Wenne et al., 2020; Zbawicka et al., 2012). Furthermore, recent multi‐locus genotyping of Scottish mussel populations using new diagnostic SNP markers identified introgressed genotypes that were not recognized by single‐locus genotyping with Me15/16 (Wilson et al., 2018). It follows that the occurrences of pure species, as revealed by single marker studies, might in some cases have been overestimated and a more precise evaluation of species composition based on multiple genetic markers would therefore be beneficial to the mussel industry. However, molecular genetic species identification is rarely accessible for mussel farm operators, who typically rely on inspections of phenotypic traits (e.g. meat yield, shell weakness and shell shape) to assess the performance of their crop and potential incidence of *MT* at their sites.

Shell phenotypic traits are influenced by many factors other than species identity or level of hybridization. The thickness and structure of a shell, which ultimately determine its strength, are affected by predation pressure (Freeman & Byers, 2006; Nagarajan et al., 2006) and by the prevailing environmental conditions, especially salinity and seawater chemistry (Almada‐Villela, 1984; Fitzer et al., 2016; Telesca et al., 2019). Similarly, shell shape is predominantly determined by shell modifications that occur during growth and which are regulated by environmental factors (e.g. temperature, salinity, food availability, wave exposure) as well as competition for space (Akester & Martel, 2000; Seed, 1968; Telesca et al., 2018). Whilst shell characters may allow for accurate species identification in allopatric populations (McDonald et al., 1991), the exposure to common environmental conditions and interspecific gene flow in sympatric populations can erode morphological differences and result in mussels of different species expressing similar shell phenotypes, that is shell plasticity (Gardner & Thompson, 2009; Innes & Bates, 1999; Seed, 1968). Consequently, it remains unclear to what extent variation in shell morphology can be explained by genetic differences, bringing into question the accuracy of species identification based solely on phenotypic traits.

In this study, we investigated the genetic structure of *Mytilus* stocks in suspended longline culture at a mussel farm in Loch Leven, Scotland, in order to determine covariance with shell phenotype over a vertical scale. Loch Leven was chosen as bathymetric and hydrographic conditions are similar to many other sea lochs on the west coast of Scotland with pronounced stratification of brackish surface water and importantly, *MT* incidence suspected by the farm operators. We used a single nuclear marker (Me15/16) and also developed and applied a new *MT*‐diagnostic SNP panel in combination with shell morphometric analyses to determine: (a) whether stock structure and the extent of hybridization with *MT* alleles varies with culture depth; (b) whether and to what extent *MT* introgression affects commercially relevant shell traits, specifically shell strength and shell shape; and (c) to evaluate whether practical shell measurements provide the potential to identify mussels with *MT* genotypes at cultivation sites. We hypothesized that *MT* introgression would be more prevalent in shallow brackish water, resulting in weaker and narrower shells, whilst mussels in deeper more saline water would have a lower proportion of *MT* alleles and stronger, more rounded shells.

## 2 MATERIALS AND METHODS

### 2.1 Study area and sample collection

**FIGURE 1:**
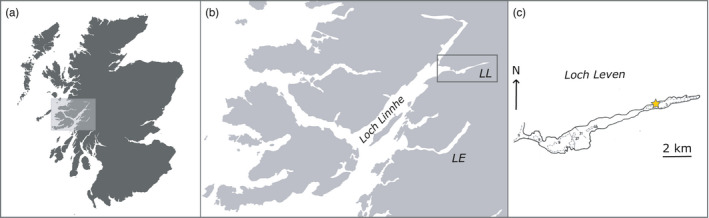
Study area and sampling location. (a) Map of Scotland; (b) inset showing the broader study area (northern Argyll and Bute, and the southern Highlands) with the sea lochs Etive (LE) and Leven (LL); (c) mussel farm location (yellow star) (56°42′34.92″ N, 5°1′43.32″ W; map adapted from Edwards and Sharples (1986)

Adult mussels (2–2.5 years of age) were collected between June 2015 and June 2016 from a longline mussel farm in Loch Leven, Argyll UK (56°42′34.92″ N, 5°1′43.32″ W; Figure 1). Loch Leven is 13.4 km long and is divided into five basins that are separated by shallow sills. The loch is relatively sheltered from all but westerly winds and receives 591.5 million m^3^ year^−1^ freshwater from the surrounding catchment (Edwards & Sharples, 1986). The tide is semidiurnal (range = 3.7 m) with restricted marine exchange, resulting in a high degree of seasonally modified stratification (Edwards & Sharples, 1986). Mussel cultivation takes place in the third and fifth basins (i.e. close to the head of the loch), where the latter is the main site of mussel production. Ten 150 m long headers are submerged in parallel at 2.5 m water depth (supported by floats), from which 4 m long droppers are strung every 0.5 m.

A total of 440 mussels were randomly collected from a series of cultivation depths: 1 m = upper mussel distribution limit (*n* = 118) [mussels growing on the support ropes], 3 m (*n* = 120) and 5 m (*n* = 119) = intermediate depths, and 7 m = lower distribution limit (*n* = 83) [end of dropper], whereby individuals were sampled from within an area of 0.5 m below and above each marked depth (e.g. 1 m = 0.5–1.5 m). Salinity *S* at each depth was recorded continuously throughout the study period (June 2015–June 2016; recording interval: every 30 min) using DST CT loggers (Star Oddi). Each mussel was processed in the following order: (i) shell strength, (ii) tissue sampling for genetic analyses and (iii) shell shape.

### 2.2 DNA extraction and genetic analysis

Mantle tissue was dissected from each individual and stored in 99% molecular grade ethanol at −20°C. Whole genomic DNA was extracted following an adapted phenol–chloroform protocol (Sambrook et al., 1989), and DNA concentration was assessed using a NanoVue Plus spectrophotometer (GE Healthcare). Each individual mussel was then identified to species level using the nuclear diagnostic marker Me15/16 (Inoue et al., 1995). Available PCR protocols (Inoue et al., 1995; modified by Dias et al., 2008 and Wilson et al., 2018) were tested and modified to achieve optimal reaction conditions. Each 20 µl PCR comprised 2 µl Taq buffer (10x), 0.4 µl dNTPs (10 mM), 0.8 µl MgCl^2+^ (25 mM), 2 µl each forward and reverse primer Me15/16 (2 µM), 0.2 µl Taq polymerase (5 U µl^−1^), 1 µl template DNA (200–300 ng µl^−1^) and 11.6 µl ultrapure water. The following PCR conditions were used: 94°C for 5 min, [94°C for 30 s, 56°C for 1 min, 68°C for 1 min] × 41 cycles, 68°C for 7 min and cooling to 4°C. PCR products (5 µl) were separated at 6 V cm^−1^ for 90 min on a low‐molecular‐weight agarose gel (4% in 1x TBE, MetaSieve, Bio Whittaker Molecular Applications) and visualized under UV light. *Mytilus* species were identified based on the size differentiation of PCR products: 180 bp (*ME*), 168 bp (*MT*), 126 bp (*MG*), with individuals showing either one (pure species) or two fragments (putative hybrids) of species‐specific size (Inoue et al., 1995).

To estimate the extent of individual introgression with *MT*, we developed a multi‐locus Fluidigm™ assay for genotyping a custom panel of SNPs that were selected a priori on the basis of them being informative about *MT* ancestry. The raw sequences used for assay design originated from a previous study (Wilson et al., 2018) that subjected pure *ME* (*n* = 21), *MG* (*n* = 15) and *MT* (*n* = 4) individuals to RAD sequencing (raw reads available at the sequence read archive, accession number: PRJEB7210). We identified a total of 134 sequences containing SNPs that were informative about *MT* ancestry and which carried either fixed differences (*MT* being homozygous for an allele that was not present in either *ME* or *MG*) or were polymorphic in *MT* but not in *ME* or *MG*. Of these, 65 loci qualified for primer design (SNP type™ assay, Fluidigm D3 software) and were used for genotyping of the mussel population, including no template controls (NTC), on a Fluidigm EP1 system with a 96.96 Dynamic Array™ (Xelect Ltd., UK). Endpoint fluorescent images were acquired for genotype calling using the SNP genotyping analysis software (Fluidigm Corp. 2016) followed by inspection of auto‐calling results and correction if required.

Genetic data analyses focussed on: (i) within‐site variation in mussel stock composition; and (ii) the magnitude of *MT* allele introgression in relation to culture depth. First, Me15/16 genotyping results were used to calculate the proportions of *Mytilus* genotypes (pure species of *ME*, *MG*, *MT* and putative hybrids *ME* × *MG*, *ME* × *MT*, *MT* × *MG*) and species‐specific allele frequencies, followed by testing for an effect of culture depth on either distribution using Fisher's exact tests. A quasibinomial GLM of weighted proportions (i.e. accounting for sample size per depth) was then used to investigate the effect of depth on *MT* genotypes. Multi‐locus SNP data were first subjected to quality control including the evaluation of each locus for its diagnostic power and the application of a 20% missing data threshold to both loci and individuals. The SNP data were then used to calculate the frequency of *MT* alleles (*MT*_AF_ = ‘0’ [no introgression] to ‘1’ [fully introgressed]), the observed heterozygosity (*H*_O_ = *nXY* (no. of individuals heterozygous for the *MT* allele)/*M* (no. of genotyped loci)) and the hybrid index (*HI* = (*nXY* + *nXX*) (sum of loci showing the *MT*‐diagnostic allele)/*M*; after Wenne et al., 2016) for each individual. *MT*_AF_ data for each individual at each locus were visualized with heatmaps (using the R package ‘*ComplexHeatmaps*’; Gu et al., 2016) together with hierarchical clustering. The relationship between *MT* allele presence (binomial response: presence ‘0’ | absence ‘1’) and culture depth (continuous predictor) was investigated using a logistic regression model (binary GLM) and calculating the odds of sighting *MT* alleles for each depth.

### 2.3 Shell strength and shape

First, mussel shell length (*SL*, mm) and shell height (*SH*, mm) (Lujii digital callipers; precision = 0.01 mm), and the total wet weight (*TWW*, g; Sartorius Quintix 124‐1S, precision = 0.1 mg) were recorded. Shell strength was then assessed in the living mussel using a custom‐built device that applies a compression force to the shell until the valve is punctured (Figure S1). For this, each mussel was placed laterally on the centre of the holding plate, with the point of maximum shell depth aligned with the compression pin (rounded tip, length = 10 cm). Subsequently, the force gauge (Sauter FK500, capacity = 500 N, resolution = 0.2 N) was lowered at a constant speed until the upper valve was punctured, and the maximum force (in kg) applied recorded, serving as a quantitative estimate of shell strength. Following mantle sampling, the remaining soft tissue was removed, the shell valves were blotted dry, and the wet weight of the shell pair (*WWS*, g) was recorded.

The data were first tested for collinearity among shell traits (predictor variables: *SL*, *SH*, *WWS*, *TWW*) via bivariate associations and using principal component analysis (PCA). Predictors that were highly correlated were removed from subsequent analysis, followed by fitting univariate regression models to investigate the relationship between shell traits and the response variable, shell strength. Shell strength data were then normalized for shell length (kg cm^−1^ *SL*), and the relationship with culture depth and the variance in seawater salinity at depth (both continuous predictors) was analysed using regression models. The relationships between the response variable, shell strength and continuous predictors, *MT*_AF_ and cultivation depth, were independently assessed using linear regression models. Model selection was performed by comparing the full model (shell strength (kg cm^−1^ *SL*) = *MT*_AF_ × depth) and reduced models using AIC scores, and selecting the model that provided the best fit of the data. Model assumptions were verified by plotting model residuals and checking for homoscedasticity and normality.

Variability in *Mytilus* shell shape was quantified using geometric morphometric analysis, following the protocol described in Telesca et al. (2018). For each mussel, shell shape was assessed on the same valve used for shell strength recordings as the procedure was nondestructive. In brief, lateral views of the right shell valve were photographed and the shell outlines marked and converted into a list of x‐y pixel coordinates (Adobe Photoshop CS4 v11.0.1). Binary images were uploaded in R (package ‘*Momocs*’; Bonhomme & Claude, 2014), and the coordinates (i.e. shell outlines) were normalized and geometrically aligned prior to elliptic Fourier analysis (EFA) (Kuhl & Giardina, 1982). Following Fourier decomposition, the number of harmonics encompassing >95% of the total harmonic power (i.e. describing most of the variation in shell shape) was chosen and the harmonic coefficients (elliptic Fourier coefficients) were extracted for each shell outline. PCA was then performed on the coefficient matrix to explore among individual variation in shell shape in relation to culture depth and level of introgression with *MT* alleles. Principal components (PCs) explaining >95% of the shape variation were retained and served as new shape variables, with each PC contributing to the variation in specific shell features. These variables were then analysed with multivariate analysis of variance (MANOVA) to quantify the effects of culture depth, genotype and other predictors (*SL*, *S*) on shape variation.

All data exploration, visualization and analysis were performed using the R software (R Core Team, 2017; v3.4.1). Results are reported as mean values with standard errors (mean ± SE). Model estimates are reported with 95% confidence intervals (95% CI), in addition to the model statistics (*F*‐value, degrees of freedom and significance values *p*‐value).

## 3 RESULTS

### 3.1 Stock composition

**FIGURE 2:**
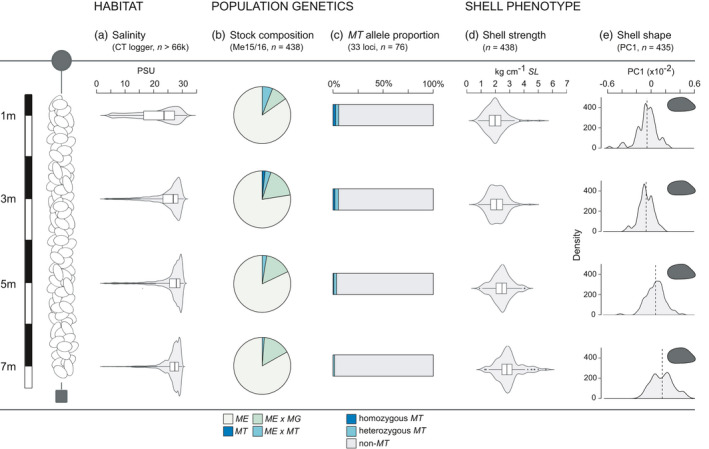
Habitat–genotype–phenotype relationships in mussels from different cultivation depths (1, 3, 5 and 7 m). *From left to right*: (a) variation in water salinity within and across culture depths; (b) relative proportions of pure and hybrid mussels of the *Mytilus* species complex (*ME*: *M*. *edulis*, *MG*: *M*. *galloprovincialis*, *MT*: *M*. *trossulus*; and putative hybrids) based on a single nuclear marker (Me15/16; Inoue et al., 1995); (c) Relative proportions of homozygous and heterozygous *M*. *trossulus*‐diagnostic alleles based on a multi‐locus SNP assay; (d) variation in shell strength (kg cm^−1^ *SL*) across depth; (e) variation in mean shell shape across depth, expressed as the distribution of PC1 values and showing the average shape per depth. Data distributions are shown as violin plots overlaid with Tukey boxplots for (a) and (d)

We genotyped 438 individuals (two individuals did not show diagnostic PCR fragments) at the Me15/16 locus to reveal four distinct genotype classes within our cultivation site. Based on this marker, *ME* was the dominant species (81.7%), followed by *ME* × *MG* hybrids (14.4%), with smaller quantities of *ME* × *MT* hybrids (3.42%) and pure *MT* individuals (0.457%). We did not find any pure *MG* individuals nor any *MG* × *MT* hybrids. The overall distribution of *Mytilus* genotypes did not vary greatly across culture depth (Fisher's exact test, *p* = 0.27; Figure 2b, Table 1), although the relative proportion of *ME* × *MT* hybrids decreased significantly by around 10% (95% CI = −0.11, −0.08) with each metre increase in culture depth (quasibinomial GLM; *ɸ* = 0.053; *p* = 0.016). Similarly, the frequency of *MT* alleles declined by 7.6% (95% CI = −12, −0.29) for each metre increase in depth (binomial GLM, *p* = 0.054).

**TABLE 1:** Species designations and allele frequencies at the Me15/16 locus in mussels in suspended longline culture

Depth	*n*	Species designation				Allele frequency		
		*ME*	*MT*	*ME* × *MG*	*ME* × *MT*	*ME*	*MG*	*MT*
1 m	118	0.85	–	0.09	0.06	0.92	0.05	0.03
3 m	120	0.78	0.02	0.18	0.03	0.88	0.09	0.03
5 m	117	0.82	–	0.15	0.03	0.91	0.08	0.01
7 m	83	0.83	–	0.16	0.01	0.92	0.08	0.01

Note

Sample sizes (*n*) are given for each cultivation depth.

Abbreviations: *ME*, *M*. *edulis*; *MG*, *M*. *galloprovincialis*; *MT*, *M*. *trossulus*.

### 3.2 *M*. *trossulus* introgression

Around half of the selected 65 *MT‒*informative SNPs did not produce reliable or readily interpretable genotypes. Five loci showed poor clustering performance, two exhibited false positives or negatives, four had greater than 20% missing data, and a further 21 produced ambiguous genotypes; these loci were polymorphic in *MT* only, but one nucleotide was shared with conspecifics (e.g. *MT* {TT, AT}, *MG*/*ME* {TT}). Furthermore, five individuals were excluded as they either failed to pass quality control (*n* = 4) or had greater than 20% missing data (*n* = 1). Consequently, our final data set comprised 33 SNPs (see Table S1 for details) genotyped in 435 individuals. All of the retained loci showed fixed differences between *MT* and *MG*/*ME* in the original RAD data and were therefore categorized as putatively *MT* diagnostic.

**FIGURE 3:**
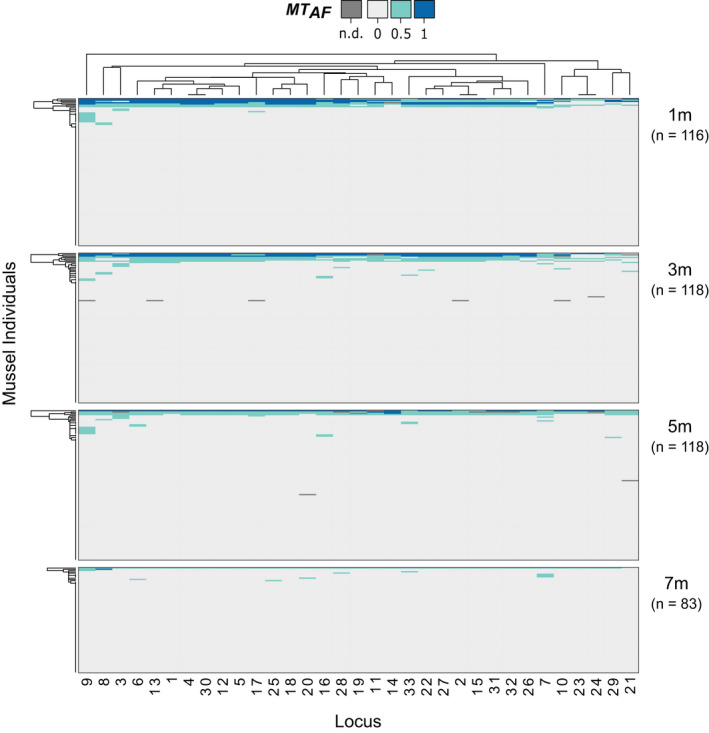
Heatmap showing the frequency of *M*. *trossulus*‐diagnostic alleles (*MT*_AF_) in mussels cultivated at different depths (columns: 33 loci, rows: 435 individuals). Individuals are hierarchically clustered according to their allele frequencies at specific loci on the x‐axis and according to their multi‐locus genotypes on the y‐axis. Dark blue cells represent homozygote genotypes for the *M*. *trossulus*‐diagnostic allele (*MT*_AF_ = 1), light grey cells represent homozygote genotypes for the nondiagnostic allele (*MT*_AF_ = 0), and light blue cells represent heterozygotes for the diagnostic allele (*MT*_AF_ = 0.5). Sample sizes (*n*) of individuals are shown for each culture depth from 1 m to 7 m

**FIGURE 4:**
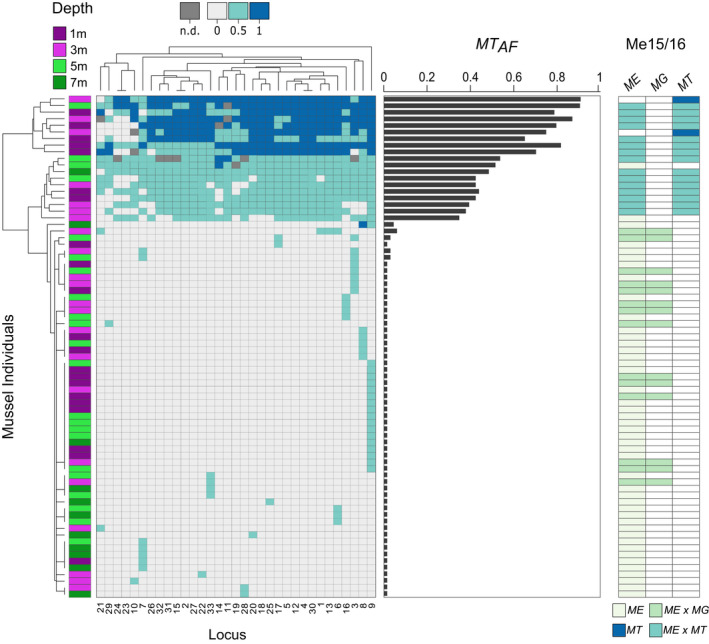
Results of the genetic analysis of 76 mussels (rows) carrying at least one *M*. *trossulus* allele. *Left*: SNP genotyping results (rows: individuals, columns: 33 loci; see legend of Figure 3 for details), *middle*: *M*. *trossulus* allele frequency (*MT*_AF_) and *right*: Me15/16 genotyping results (alleles of *M*. *edulis ME*, *M*. *galloprovincialis MG*, *M*. *trossulus MT*; pure species and putative hybrids)

Most mussels (*n* = 359; 82.5%) did not carry any *MT*‐diagnostic alleles (*MT*_AF_ = zero, i.e. 100% allelic contributions from *ME* and/or *MG*). However, 76 individuals (17.5% of the sampled population) carried at least one *MT*‐diagnostic allele (Figures 2c and 4, and Table 2). Both the proportion of individuals carrying *MT*‐diagnostic alleles and the overall frequency of *MT*‐diagnostic alleles (*MT*_AF_) were highest in surface‐grown mussels and declined with increasing depth (Table 2; also note the steady decrease in the hybrid index *HI*). PCA of the SNP data set revealed three main clusters which corresponded to individuals carrying different proportions of *MT* alleles (see Figure S2). Although logistic regression analysis showed no evidence for an overall effect of culture depth (binary GLM of *MT* presence/absence ~depth: *df* = 1, 431, *p* = 0.56), the odds of finding mussels with allelic contributions of *MT* declined by a factor of 0.96 (95% CI = 0.86, 1.08) with each metre increase in depth (odds of sighting = 22.9% at 1 m and 18.7% at 7 m depth).

**TABLE 2:** Level of introgression with *M*. *trossulus* alleles in mussels in suspended longline culture

Depth	*n*	*MT*‐positive	*MT* allele frequency (*n*)				Summary statistics		
		% of population	<10%	10–50%	50–75%	>75%	*MT*_AF_	*H*_O_	*HI*
1 m	116	18.1	14	2	2	3	0.042	0.032	0.058
3 m	118	18.6	15	4	1	2	0.037	0.035	0.055
5 m	118	18.6	18	1	2	1	0.023	0.030	0.038
7 m	83	13.3	10	1	–	–	0.008	0.015	0.016

Note

*MT*‐positive: mussels with allelic contributions of *M*. *trossulus*. Summary statistics: *MT*_AF_ = *M*. *trossulus* allele frequency, *H*_O_ = observed heterozygosity, *HI* = hybrid index (see Methods).

Detailed inspection of the ‘*MT*‐positive’ mussels (i.e. carrying at least one *MT*‐diagnostic allele, *n* = 76) revealed several patterns (Figure 4). First, 11 individuals (14.5%) had *MT*_AF_ values above 0.5 and most of these mussels were predominantly homozygous for *MT*‐diagnostic alleles. Second, eight individuals (10.5%) had *MT*_AF_ values between 0.35 and 0.5 and were predominantly heterozygous for *MT*‐diagnostic alleles, suggestive of first‐generation hybrids. The remaining individuals carried *MT*–diagnostic alleles at just one or a small handful of loci. Finally, the multi‐locus results were reasonably concordant with stock designations based on the Me15/16 locus. In particular, the majority of mussels with *MT*_AF_ > 0.3 were identified either as pure *MT* or as *MT* hybrids at the Me15/16 locus, whereas no individual with *MT*_AF_ < 0.1 tested positive for *MT* using the Me15/16 assay. However, the Me15/16 locus only identified two individuals as pure *MT* and failed to detect any *MT* ancestry in two individuals with *MT*_AF_ values of 0.35 and 0.5, respectively.

### 3.3 Shell strength

Mussel shell strength varied markedly within the cultured stock, with shell valves resisting between 3.95 kg and 31.7 kg of compression force prior to shell puncture. Shell strength was mainly influenced by shell weight *WWS* (*F*_1, 436_ = 409, *p* < 0.001) and shell length *SL* (*F*_1, 435_ = 81.9, *p* < 0.001) and followed a positive allometric relationship with either parameter. Shell strength, normalized for shell length, differed significantly across depth, being lowest for mussels at 1 m (2.03 ± 0.69 kg cm^−1^ *SL*) and increasing by 0.14 kg cm^−1^ *SL* (95% CI = 0.113, 0.173) with each metre increase in culture depth (*F*_1, 435_ = 87.3, *p* < 0.001). At the same time, shell strength followed an inverse relationship with the salinity of the seawater, with mussels experiencing large variation in salinity tending to have weaker shells and vice versa (*F*_1, 435_ = 68.6, *p* < 0.001; see Figure 2a,d).

We further explored the relationships between shell strength, *MT* introgression and cultivation depth. Both predictors significantly contributed to the variation in shell strength (shell strength (kg cm^−1^ *SL*) = *MT*_AF_ + Depth; *F*_2, 427_ = 60.5, *p* < 0.001). Shell strength decreased by 1.37 kg cm^−1^ *SL* (95% CI = −1.86, −0.886) in the presence of *MT* alleles and increased by 0.135 kg cm^−1^ *SL* (95% CI = 0.105, 0.164) with each metre increase in depth.

### 3.4 Shell shape

**FIGURE 5:**
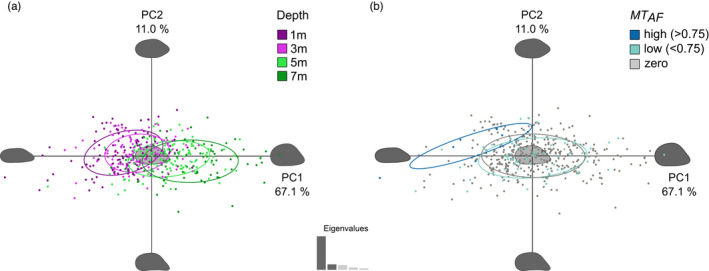
Variation in *Mytilus* shell shape, following PCA performed on elliptic Fourier coefficients of lateral views of the right shell valves. The variation explained by the first two principal components (PC1 and PC2) is given as percentages, showing the variation in PC scores for the sampled population (*n* = 435) as a function of (a) depth and (b) frequency of *M*. *trossulus* alleles (*MT*_AF_). Individuals are colour‐coded per group (see colour legends), and the 80% confidence intervals (ellipses) of each group are shown to visualize the separation among individuals across the morphospace. Reconstructed shell outlines at the extremes of the morphospace are shown along each PC axis

Shell outlines were extracted for 435 individuals (five shell pairs were highly deformed and were therefore excluded), and variation in shell shape was investigated with respect to culture depth and genotype. The first two PCs, resulting from PCA of the harmonic coefficients (*n* = 7 harmonics; 99% of total shape information), accounted together for 78.1% of the shape variation among individuals (Figure 5). Individual PCs captured the variation in one or more specific shell features, with PC1 (67.1%) mainly describing variation in shell height and ligament length and PC2 (11.0%) describing variation in the anterior margin and ventral line (see Figure S3). Individuals predominantly separated along the first axis, which described a gradient from slender and elongated shells with low PC1 values to wider and rounder shells with high PC1 values (Figure 2e). Differences in shell shape were found in relation to both culture depth and genotype. Shallow‐water mussels showed a clear tendency for dorso‐ventral compression, which progressively declined with increasing depth (Figure 5a). When individuals were classified according to their level of *MT* introgression (*MT*_AF_ > 0.75 = ‘high’, *MT*_AF_ < 0.75 = ‘low’ and *MT*_AF_ = zero ‘none’), there was also a clear separation along the first axis, with highly introgressed mussels forming a distinct cluster characterized by elongated shells (Figure 5b). MANOVAs performed on the first five PCs (>95% of shape variance explained) revealed significant effects of both culture depth (*Wilk's Λ* = 0.56, *approx*. *F*_1, 424_ = 67.1, *p* < 0.001) and *MT*_AF_ (*Wilk's Λ* = 0.85, *approx*. *F*_1, 419_ = 15.2, *p* < 0.001) on shape variance. Pairwise MANOVA further showed that depth‐specific shell shapes differed significantly from one another, with differences being most pronounced between shells from 1 m and 7 m (approx. *F* = 50.8) and least pronounced between shells from 1 m and 3 m (approx. *F* = 4.5).

### 3.5 Identification of *MT* using shell characters

**FIGURE 6:**
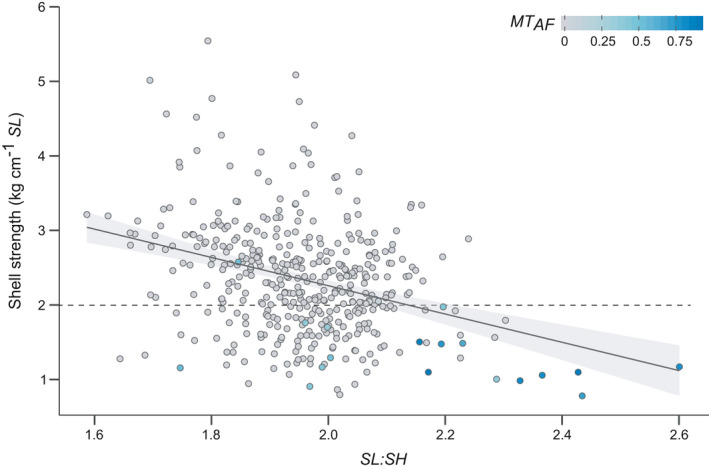
Relationship between shell strength (kg cm^−1^ *SL*) and the ratio of shell length *SL* to shell height *SH* (as a proxy for shell shape) in mixed‐species culture of *Mytilus* (*n* = 431). Individuals are colour‐coded according to their frequency of *M*. *trossulus* alleles (see legend; *MT*_AF_ = ‘0’ – no introgression to ‘1’ – fully introgressed, i.e. pure *MT*). The majority of *MT*‐introgressed mussels had a shell strength of <2 kg cm^−1^ *SL* (dashed line)

Finally, we determined the relationships between shell strength, shell shape and mussel genotype to explore the potential of simple morphological measurements to identify pure *MT* or heavily introgressed individuals within mixed‐species populations. For this, we used the ratio of mussel shell length to height (*SL*:*SH*) as an indicator of shell shape, with high values representing more elongated shells. Shell strength decreased by 1.91 kg cm^−1^ *SL* (95% CI = −2.42, −1.39) for an increase in one in the *SL*:*SH* ratio (Figure 6; *F*_1, 435_ = 52.9, *p* < 0.001). Heavily introgressed individuals (*MT*_AF_ > 0.75) had some of the lowest shell strengths and among the most elongated of all shells (strength <2 kg cm^−1^ *SL* and *SL*:*SH* >2). Significant effects of both *SL*:*SH* and *MT*_AF_ (both *p* < 0.001) were found on shell strength (*F*_2, 427_ = 33.1; the interaction *term SL*:*SH × MT*_AF_ *was* not significant, *p* = 0.16).

## 4 DISCUSSION

The increased prevalence of *ME* × *MT* hybrids at Scottish aquaculture sites led Beaumont et al. (2008) to suggest that suspended longline cultivation may facilitate introgressive hybridization, ultimately negatively impacting the Scottish mussel industry. Since environmental conditions differ across depth, longline cultivation is hypothesized to offer multiple environmental niches open to exploitation by various mussel species and their hybrids. To test whether longline cultivation leads to the vertical partitioning of genotypes, we combined a species‐diagnostic marker with a novel *MT*‐diagnostic multi‐locus SNP assay to determine the covariance between mussel genotype and shell phenotype down the length of a highly stratified (Figure 2a) cultivation rope in Loch Leven on the Scottish west coast. Despite *ME* being the dominant species across all depths, significant numbers of pure *MT* individuals as well as *ME* × *MT* hybrids were detected. Furthermore, the presence and frequency of *MT* alleles varied down the rope, with most heavily introgressed individuals being more abundant in the surface waters. Additionally, *MT* introgression explained a significant proportion of the variation in commercially relevant shell characters within a mixed‐species culture, where the most relevant trait, shell strength, can be readily and cheaply assessed. We discuss the implications of these findings for the Scottish mussel industry.

### 4.1 Stock composition

Overall, over 96% of the sampled stock from Loch Leven comprised either pure *ME* individuals or *ME* × *MG* hybrids, whilst less than four per cent had detectable *MT* ancestry. These proportions are consistent with a survey of 41 mussel farms around Scotland, which documented *MG* alleles at the majority of sites and only a few farms with *MT* alleles (Dias, Dordor et al., 2009). The presence of *MG* in Scottish waters is unlikely to have negative implications for the local mussel industry (Dias, Dordor et al., 2009) given their widespread distribution in British waters (Skibinski et al., 1983) and the well‐established cultivation of this species in Europe, particularly in Spain (FAO, 2018).

By contrast, the increased occurrence of weak‐shelled mussels with detectable *MT* ancestry has had significant economic effects on the regions concerned (Gubbins et al., 2012). Our study found negative associations between culture depth and both the proportion of *ME* × *MT* hybrids and the frequency of *MT* alleles. Given that these trends strengthened when *MT* introgression was quantified more precisely using our multi‐locus SNP assay, we conclude that stock structure does vary with depth, but that our sample size does not offer sufficient power to detect an overall pattern using a single diagnostic marker given the relatively low overall frequency of *MT* alleles.

Beaumont et al. (2008) previously found evidence for *MT* introgression in mussels from nearby Loch Etive. Both Lochs Leven and Etive are hydrographically connected to the Firth of Lorn‐Loch Linnhe estuarine system (Figure 1), have a long and slender shape and a large catchment area and are affected by high freshwater run‐offs resulting in reduced surface salinities, which is often related to *MT* occurrence (e.g. Kijewski et al., 2006; Riginos & Cunningham, 2005; Theisen, 1978). Nevertheless, our results show that *MT* alleles are considerably less abundant in Loch Leven (3.4%) than in Loch Etive (22%; Beaumont et al., 2008). The reason for this is unclear, partly because we lack an understanding of how *MT* became established in Scotland in the first place. One possibility is that the Loch Etive population could be a postglacial relict (Beaumont et al., 2008; Zbawicka et al., 2010). Alternatively, *MT* alleles might have been introduced more recently via larvae or spat transported in ballast tanks or via spat movements between cultivation sites (pers. comm. Association of Scottish Shellfish Growers; Stirling & Okumus, 1994). Furthermore, environmental heterogeneity, contrasting selective pressures, neutral drift during population bottlenecks or differences in aquaculture practices could all potentially have contributed towards the observed geographical distribution of *MT* alleles.

### 4.2 *M*. *trossulus* introgression

Efforts to develop a panel of *MT*‐diagnostic SNPs were successful, with approximately half of the oligonucleotide pairs generating clearly interpretable polymorphic genotypes with low rates of missing data. High failure rates of 60% or more are common when developing SNP assays (Helyar et al., 2011) and occur as a result of interpreting sequencing errors as SNPs, designing probes that inadvertently span intron–exon boundaries, and calling SNPs in contigs assembled from paralogous genes (De Wit et al., 2015; Helyar et al., 2011; Milano et al., 2011; Wang et al., 2008). By mapping the *Mytilus* RAD sequences to a *MG* reference genome, we were able to control for the genomic context. However, the discovery panel of *MT* individuals used by Wilson et al. (2018) was rather small, which may explain why only a limited number of *MT*‐diagnostic SNPs were available for assay design.

Overall, there was good concordance between the results of the Me15/16 locus and our multi‐locus SNP assay. Specifically, we were able to confirm that most mussels lacked *MT*‐diagnostic alleles and only carried *ME* and/or *MG* allelic contributions. However, the Me15/16 locus failed to detect any *MT* ancestry in two individuals with substantial proportions of *MT*‐diagnostic alleles (*MT*_AF_ = 0.35 and 0.5 respectively), and further classified only two individuals as ‘pure’ *MT* despite several others having comparable *MT*_AF_ scores, implying similar levels of introgression. These differences are not necessarily surprising because the diagnostic power of a single marker is limited when assessing levels of introgression (Twyford & Ennos, 2012; Wilson et al., 2018). Overall, our results suggest that there may be little to gain by deploying large panels of genetic markers for screening mussels for introgression in an industrial setting, although occasional false negatives are to be expected with a single diagnostic marker (Weissensteiner & Lanchbury, 1996).

Arguably the greatest difference between the two methodologies was that the SNP assay classified 17.5% of the mussels as carrying one, or only a very small number of *MT*‐diagnostic alleles. Although this may be interpreted as evidence for low levels of background introgression with *MT*, it is more likely that not all of the SNPs are strictly diagnostic given the relatively small discovery pool of individuals in the original RAD sequencing study (21 pure *ME*, 15 pure *MG* and four pure *MT* mussels). Sequencing a total of 72 *ME*/*MG* chromosomes should, in principle, allow the detection of alleles down to a minor allele frequency (MAF) of approximately 1.4%. However, this may be higher if not all of the chromosomes were sequenced to an equal depth of coverage. Consequently, it is possible that at least some of the alleles classified as being fixed in *MT* might be present at low frequencies in *ME*/*MG*, which may explain why several mussels scored positive for a single *MT* allele, often at the same small subset of loci (see Figure 4). Moreover, screening only eight *MT* chromosomes provides little power to detect allelic variants below a MAF of approximately 12.5%. Consequently, where *ME*/*MG* genotypes are observed in mussels with high *MT* genomic contributions, it is possible that these are false negatives for *MT*. Loci showing this pattern were infrequent in our data set (see the top left portion of the heatmap in Figure 4) but they may have resulted in *MT* introgression being slightly underestimated.

Overall, our results suggest that the magnitude of *MT* introgression (measured by *MT*_AF_, *H*_0_ and *HI*; see Table 2) was higher for mussels cultivated at shallow depths. Environmental cultivation conditions varied more in surface waters than at greater depth and shallow‐water mussels were exposed to lower and highly variable seawater salinities (see Figure 2a). Current and previous observations (Beaumont et al., 2008; Dias, Bland et al., 2009) suggest that *MT* alleles occur more frequently under these conditions consistent with *MT* adaptation to low‐salinity environments, where it potentially outcompetes the less freshwater tolerant *ME* (Qiu et al., 2002; Ridgway & Nævdal, 2004; Zbawicka et al., 2014). The current findings raise the question of how this variation in stock structure along the cultivation rope could become established. Studies of comparative settlement depths of mixed‐species populations of *ME* and *MT* in Canada showed that both species have different settlement patterns with respect to depth, which can be further modified in the presence of a thermocline, that *ME* is found more frequently at greater depth than *MT*, and that this species‐specific settlement preference persists through time (Freeman et al., 2002; Kenchington et al., 2002).

### 4.3 Shell plasticity

Building on the work of Beaumont et al. (2008), we developed a low‐cost device for quantifying shell strength in living bivalve molluscs that can be easily assembled and transported for use by scientists and farm operators alike. We found that *Mytilus* shell strength varied considerably with culture depth, with weak‐shelled mussels being present almost exclusively at shallow depth, whereas stronger shelled mussels predominated at greater depth. This vertical distribution may reflect the pronounced environmental heterogeneity at the study site, specifically low and variable surface salinities (Figure 2a) which may have contributed towards the lower average shell strength of shallow‐water mussels. This is consistent with the observation that *Mytilus* spp. from low‐salinity environments exhibit significant reductions in shell dry weight and calcareous content (Kautsky et al., 1990; Telesca et al., 2019). Depending on the extent and duration of low‐salinity exposure, this may limit shell growth and induce internal shell dissolution (Almada‐Villela, 1984; Grenier et al., 2020) making mussels weaker and more vulnerable to physical stressors and predation (Nagarajan et al., 2006).

Our results further showed that a mussel's shell strength is not only influenced by the cultivation environment (i.e. depth) but is also partially under genetic control, with shell strength decreasing with increasing proportions of *MT* alleles. This provides further evidence in support of Beaumont et al. (2008) more qualitative findings of significantly reduced strength in fragile‐shelled mussels.

Geometric morphometric analysis of shell outlines revealed marked variation in shell shape with cultivation depth, from elongated and narrow to round and wide mussel shells with increasing depth (see Figures 2e and 5a). This transition in shape is consistent with a recent study showing that salinity influences shell shape over a variety of spatial scales (Telesca et al., 2018). Furthermore, mussels with *MT* allelic contributions of >75% had distinctive elongated and dorso‐ventrally compressed shells. Previous studies of *Mytilus* shell shape have shown that mussels typically cluster by location irrespective of their genotype when macrogeographical scales are considered (e.g. Europe versus North America; Gardner & Thompson, 2009), probably due to the overriding effects of variation in biophysical conditions. Conversely, clear associations between genotype and shell shape have been found at smaller spatial scales (e.g. along the coast of Newfoundland; Penney & Hart, 1999). Our study focused on a single site, effectively controlling for geographic effects and macrogeographic population genetic structure. The discovery of a relationship between mussel genotype and shell shape in the current study therefore supports the argument that shell morphology is partly under genetic control in *Mytilus*.

### 4.4 Implications for mussel cultivation

Scottish coastlines offer many locations that are suitable for molluscan mariculture, since they are often shallow, sheltered and associated with moderate water flow. However, hydrological differences between sites influence farm designs and cultivation practices. This study builds upon previous evaluations of suspended mussel culture in Loch Leven (Okumuş, 1993; Okumuş & Stirling, 1998; Stirling & Okumuş, 1994) by identifying the presence of *MT* alleles and their association with shallow cultivation depths. Although both pure *MT* and *ME* × *MT* individuals were found, our results are specific to one locality and sampling time. As seawater conditions vary over spatial and temporal scales, we may expect that mussels sampled from the same or other locations and at different time points will differ in their genotype proportions. Consequently, we advocate further monitoring of Loch Leven as well as other Scottish production sites with comparable sea loch features and/or the suspected presence of *MT* (e.g. lochs Fyne, Eil, and Sunart; Edwards & Sharples, 1986; Gubbins et al., 2012).

Our findings suggest that shell strength can serve as a useful indicator of stock performance and *MT* presence. In comparison with molecular species identification, shell strength quantification is rapid, cost‐effective, can be performed on site and directly focuses on shell characters that are important for commercial cultivation. We also show that the ratio of shell length to height (*SL*:*SH*) can provide a simple indicator of mussel genotype, especially when used in combination with shell strength. Species‐specific differences in *SL*:*SH* have been reported in Canadian mixed‐species populations of *Mytilus* and allowed for the identification of *MG* and *MT* (Elliott et al., 2008). However, these authors also noted that the underlying morphometric equation may differ across locations and geographic areas, thus again acknowledging an environmental influence on shell morphology.

Finally, an improved understanding of environment–genotype–phenotype relationships in cultivated *Mytilus* reveals several potential mitigation strategies. For sites like Loch Leven, it may be beneficial to collect mussel spat from different locations within the sea loch that are more exposed to the open sea, where the water column is generally well‐mixed, and the influence of freshwater run‐off is minimized. This practice should favour the settlement of *ME* and thereby reduce the risk of introducing *MT* alleles into the commercial stock. Given that we also found clear evidence for weaker shells and higher levels of *MT* introgression at shallow culture depths, producers should consider deploying droppers at greater depth (i.e. below the halocline) to minimize the influence of the low‐salinity surface layer that favours *MT* (a depth greater than 3 m is suggested for Loch Leven). Finally, spat movements between lochs should be avoided as these may inadvertently introduce *MT* into previously unaffected areas.

## 5 CONCLUSIONS

Our study complements previous work on *Mytilus* species composition and hybridization on regional scales as well as between shoreline and rope‐cultured populations by providing new insights into genetic and morphological variation on a very fine spatial scale: along a cultivation rope. Environmental heterogeneity within culture habitats can impact stock structure, highlighting the importance of understanding interrelationships among environmental, genetic and phenotypic parameters. Finally, we point towards potential practical solutions for mitigating the further spread of this commercially damaging species in Scotland, which may help to better inform culture practices and ultimately increase the productivity, capacity and sustainability of local mussel farming operations.

## CONFLICT OF INTEREST

None.

## Supporting information

Supplementary Material

## Data Availability

The data that support the findings of this study are openly available in ‘figshare’ at https://doi.org/10.6084/m9.figshare.14333960.v1
