# Supplementary material for: Mytilus trossulus introgression and consequences for shell traits in longline cultivated mussels
Source: Evol Appl. 2021 May 10;14(7):1830–43. doi: 10.1111/eva.13245 (PMC8288009; doi:10.1111/eva.13245)
Supplement: Supplementary file 1 — Supplementary Material [file EVA-14-1830-s001.docx]

**Supplementary Material**

**Figure S1** Custom-built device for quantitatively measuring shell strength in mussels. The design was adapted from Penney et al. (2007) and Beaumont et al. (2008) and the apparatus was manufactured in house (SAMS). See section 2.3 for a description of the mode of operation.


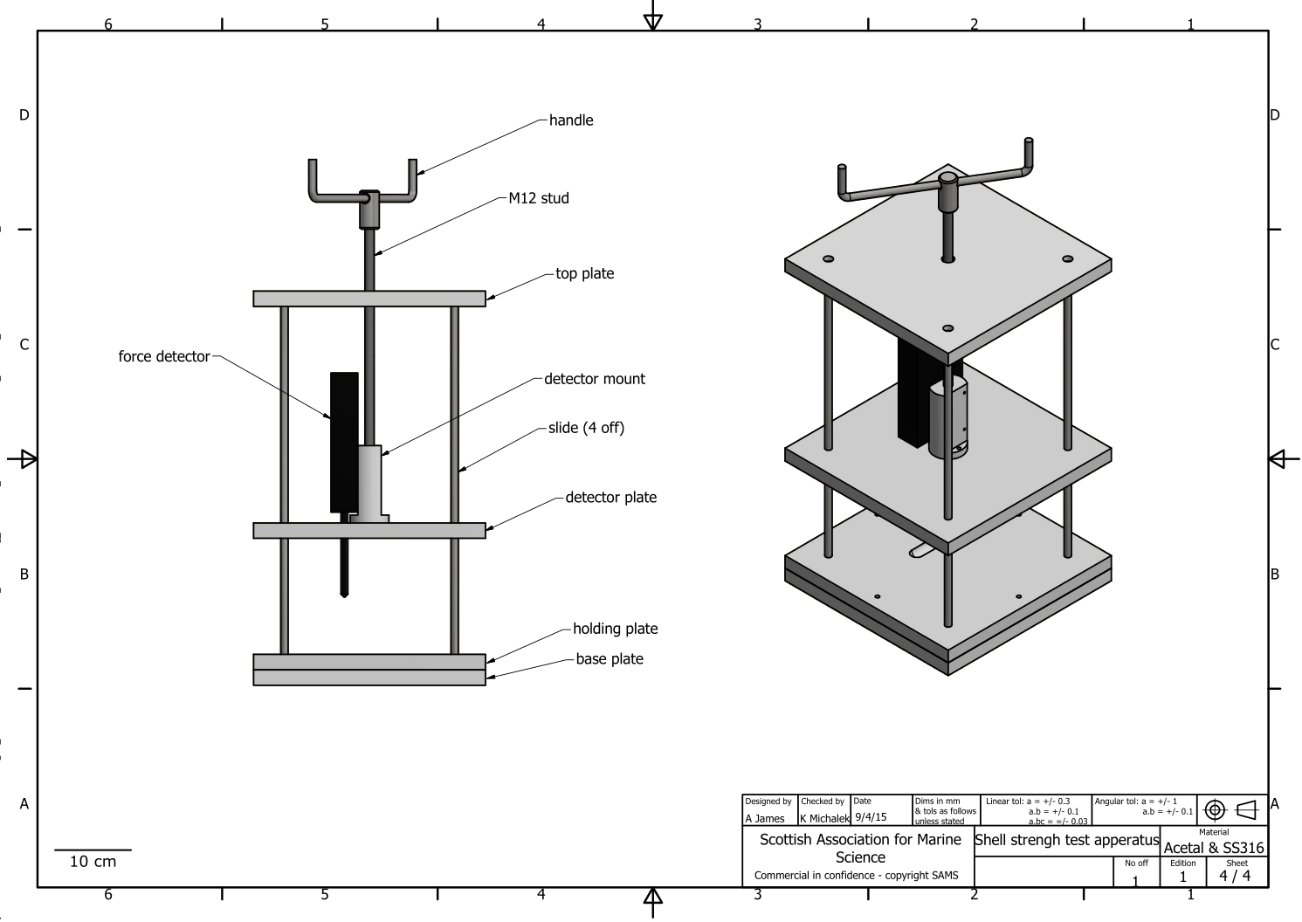


**Figure S2** PCA biplot of the SNP allele frequencies (*n* = 33 SNPs) in cultured mussel from Loch Leven, Scotland (*n* = 435 individuals); colour-coded by their depth of cultivation. PC1 captured most of the genetic variation, with three main clusters being observed and corresponding to the individuals carrying different relative proportions of *MT* alleles. *From left to right*: mussels with none or low, intermediate, and high *MT* allele frequencies (*MT_AF_*), respectively.


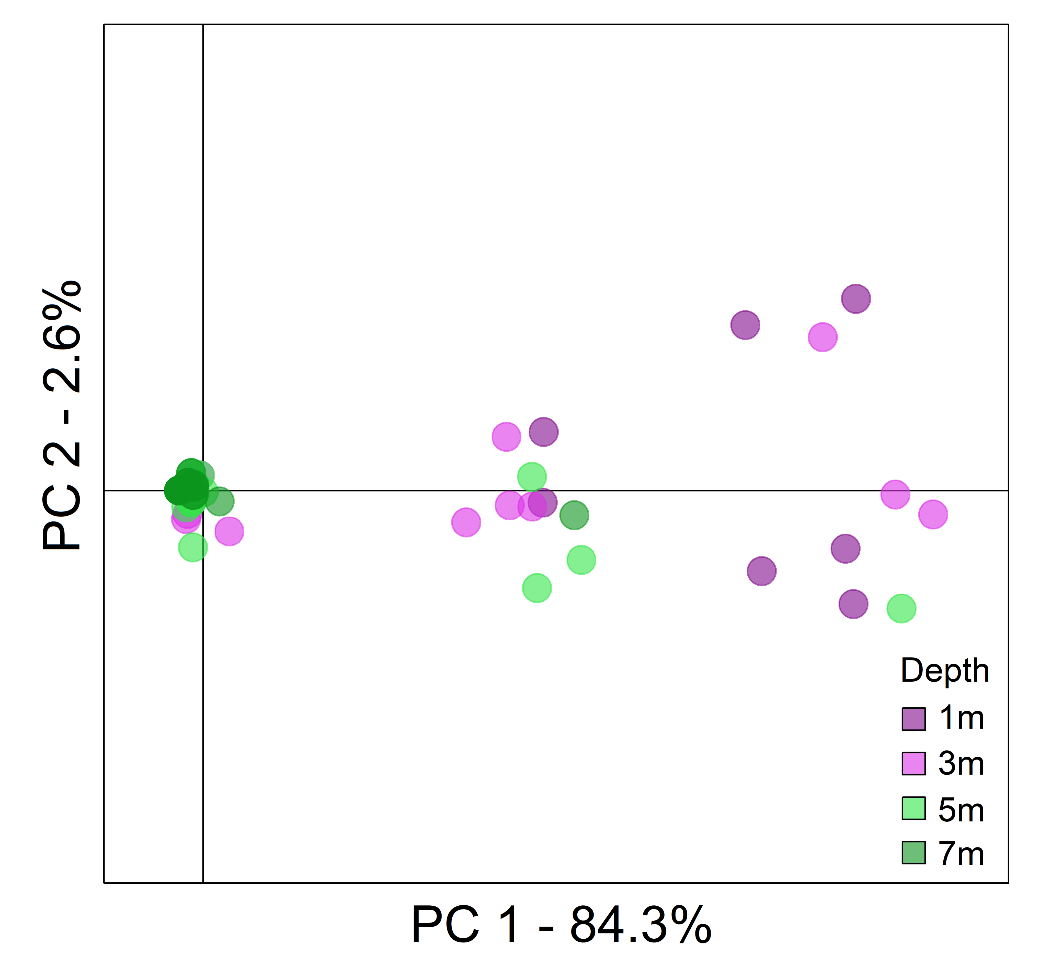


**Figure S3** Overview of the first five principal components (PC1-5; ‘shape variables’) capturing more than 95 % of the variation in *Mytilus* shell shape. *From left to right* (for each PC): (a) The percentage of variance explained, the contribution to shell outline reconstruction with decreasing / increasing PC values (mean PC value ± 3 standard deviations), and to specific shell features. (b) The effect of selected predictors (‘*SL*’ shell length, in mm; ‘*MT_AF_*’ frequency of *M. trossulus* alleles, 0 - 1; ‘*S*’ salinity, in PSU) on each PC. Loess-smoothed curves to aid the visualisation of individual relationships. Univariate GLMs revealed significant effects of some predictor variables on each PC (*p*-value < .001 ‘***’, < .01 ‘**’, < .05 ‘*’).


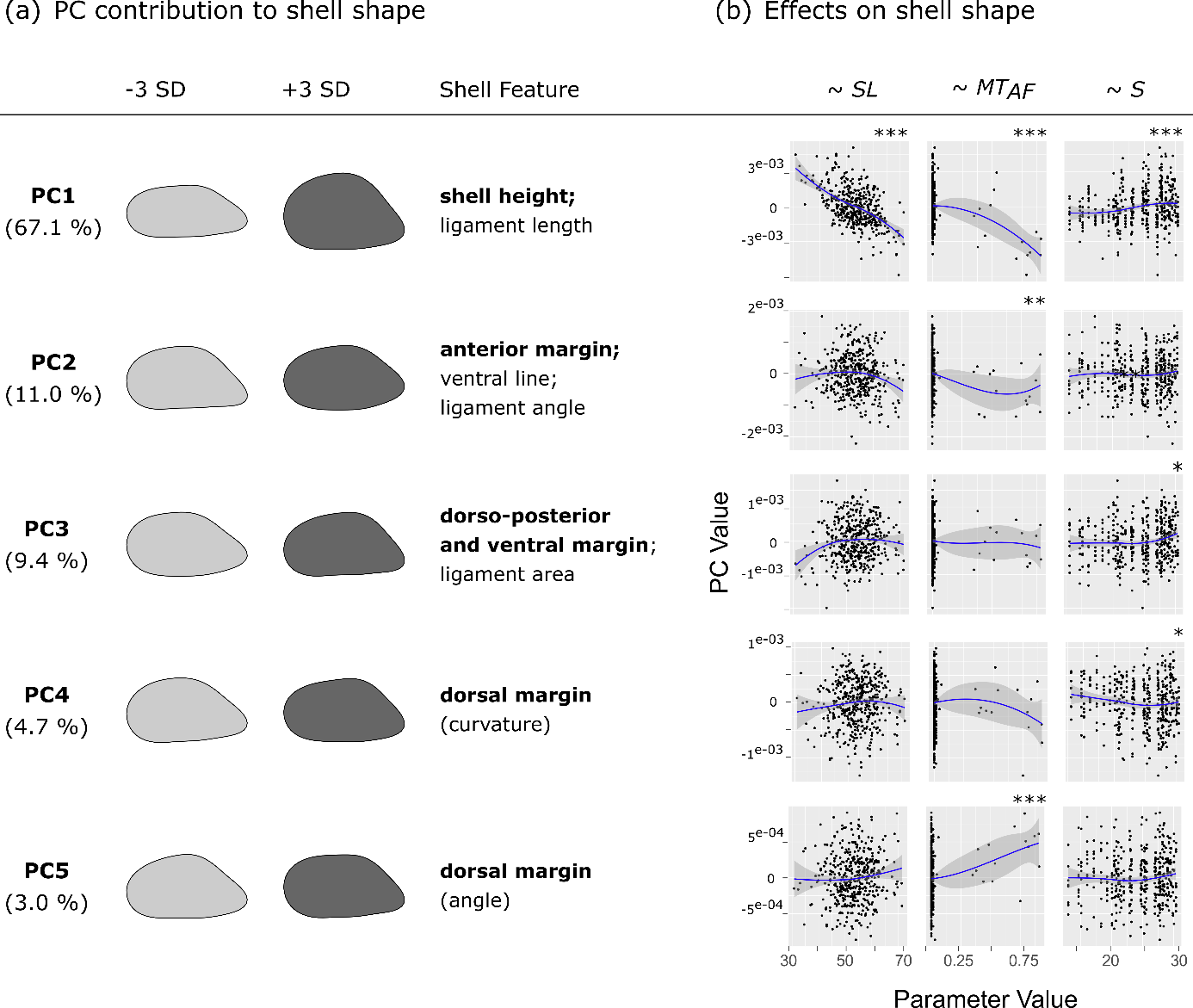


**Table S1** SNP marker panel (n = 33) diagnostic for *Mytilus trossulus* (MT). Shown are the locus ID and reference, species-specific nucleotide call, and RAD sequence for each marker. The number of genotyped mussels (sample size, n) and the number of individuals showing alleles of MT (MT-positive), also expressed as proportion of the genotyped sample population, are shown for each marker, respectively.

| **Locus** | **Reference** (Primer; Assay) | **Allele** |  |  | **RADseq marker sequence** | **Sample Size** | **MT-positive** | |
| --- | --- | --- | --- | --- | --- | --- | --- | --- |
|  |  | ME | MG | MT |  | n | n | % Pop |
| 1 | 100173_B GTA0189062 | {TT} | {TT} | {AA} | TGCAGTTTCTTGATAAGCAAATAGTTGATATGACTG[AT]ATTTCTGCATGATTTTTAGCAATATAGGTCATATTCCATGATAAAACAATGCCTAC | 435 | 19 | 4.37 |
| 2 | 117000_B GTA0189052 | {TT} | {TT} | {GG} | TGCAGAGAAGTTAAAGTTTTGTCCAGGTGGAGAAGTCATTGA[GT]GCTGAATGTGCAACCAAAGATGGCATGCCTTATGATGGTACTGGTGAGAT | 434 | 19 | 4.38 |
| 3 | 24411_A GTA0189075 | {AA} | {AA} | {GG} | TGCAGCTTTCAAAAAGGAATCTGGTTTATTCGATTCA[AG]TGAATGTTACCCCTACTATATGACTGCTATGGTTTGCTCAATATTTTGTTATTTA | 434 | 23 | 5.30 |
| 4 | 39506_A GTA0189092 | {AA} | {AA} | {TT} | TGCAGATGCAAAAGATAGAGCAAAAAGAATATTGGCAGGTTGTAGAGGAGG[AT]AGTATTGGGTCTTACACTGCTAGCCCTGGTGTTGAGGTTAT | 435 | 19 | 4.37 |
| 5 | 76606_B GTA0189093 | {TT} | {TT} | {AA} | TGCAGATCTGGTTAGTAACACAAGGG[AT]TTTACAAAGCAAACATTTAGCTATATTAATTATATTTGTTTGAAGAGAAAACCATGAAATTGACAA | 435 | 19 | 4.37 |
| 6 | 100259_A GTA0189097 | {AA} | {AA} | {GG} | TGCAGCCGAATTAGAAACTATTAAAAAGAAAATGGA[AG]GGAGAGAATGATGAGCTCAAGAAAGATATAGAAGATTTAGAAAGTTCTCTTGCAAA | 435 | 23 | 5.29 |
| 7 | 123343_A GTA0189068 | {GG} | {GG} | {AA} | TGCAGCTTCAGCTTCTTCACGTTTCTTCTTTTCTTCCTCTTCCTGGTCCAATTCTTTGAT[AG]CTTTCTTCTAAAACATTGGGCCAGAAATCTCC | 435 | 22 | 5.06 |
| 8 | 141978_A GTA0189090 | {GG} | {GG} | {AA} | TGCAGATTAAACGCCAACCAAAACAGAAATTAAAGCCAAA[AG]CGTTATTGTCTCATTATTGGAATTAATCAGGATTTTATTAAAATATAAATAT | 435 | 23 | 5.29 |
| 9 | 212387_A GTA0189078 | {AA} | {AA} | {CC} | TGCAGATTACATAATATCTCTGCACTTGATGTAACCCATAAAACATT[AC]AAATGAACCCTGTCATTACTGCTCTGACATTTATATAGCCCTCCT | 434 | 37 | 8.53 |
| 10 | 123488_A GTA0189044 | {AA} | {AA} | {CC} | TGCAGCAGCCTCGTTTCCATATGG[AC]ATACCCTCTGCAAATTCTAGTCCATTTAATGTATACTCACAAATGGGCATCCAAAGACCAACAAACAA | 432 | 14 | 3.24 |
| 11 | 26598_A GTA0189045 | {AA} | {AA} | {TT} | TGCAGACTGTATGTAATGTTAACTGGAAGAAAAAATATC[AT]GAGTCCATTTATAAGTAATATATACATGTATGTTTAAACAGGAAATATTTTTT | 433 | 17 | 3.93 |
| 12 | 53148_A GTA0189071 | {CC} | {CC} | {TT} | TGCAGCATCATAATCATATAAAACTCTAGC[CT]TGTTTTTTCTCTGTACTAGGTGTTGCAGCTGTGATGTGTATCATAGGGGGTGCACTTGGCTG | 435 | 19 | 4.37 |
| 13 | 8289_B GTA0189070 | {AA} | {AA} | {AC,CC} | TGCAGATTGACCAGCTATTGTTATAGAATTTGGATTCCCTCCAAAACTTTCAATATTGTCATG[AC]ACCCATTTAATCGCCATTTGTTGGTCCCA | 434 | 20 | 4.61 |
| 14 | 10704_A GTA0189058 | {CC} | {CC} | {TT} | TGCAGATCATATTTTCACTATTAAAACTTTGATAAATAAATATTTACA[CT]AAATTAAAAAAGCCTATTTTTGCTTGTTTTGTAGATCTCTCTAA | 434 | 15 | 3.46 |
| 15 | 33513_A GTA0189101 | {TT} | {TT} | {GG} | TGCAGATGCAATTACTTCTAAATGTGGATGCCACACAAAGATAATT[GT]CACACAACATCCCTAATTAATTTTGTTTTCTCTTGTAGAACATGCT | 434 | 18 | 4.15 |
| 16 | 84029_A GTA0189049 | {CC} | {CC} | {TT} | TGCAGGCAGGGGATGGTATCCTATT[CT]ATTAACAATGCCAACACAGATCAGCTATCACATGAACAAGCTAAAATGGAGATGATCAGATCTGGCA | 435 | 21 | 4.83 |
| 17 | 108748_B GTA0189082 | {CC} | {CC} | {TT} | TGCAGTAATGGACCTTGCTTCCTTTGCCGCTTCCATTGCAAA[CT]GATACTGCTAATTTAACGCAAATGACAATATCTCCTACAAATTTGGATGC | 434 | 21 | 4.84 |
| 18 | 131765_A GTA0189100 | {TT} | {TT} | {GG} | TGCAGGATGCACTTGATATAGAAAACATGTCTCGTTAGGTTATAATCTATGTA[GT]AATCCAAATAACGTGGAATCAACATGCATATACGCAAAA | 435 | 18 | 4.14 |
| 19 | 187177_A GTA0189085 | {GG} | {GG} | {TT} | TGCAGATGGGTTTGAATACTGGA[GT]TATCTTTGTCATTTCCTGTGCTGTTTTGTGCACTGTGAATATATTGTCCTGACCGAGAACTACGAAATC | 434 | 15 | 3.46 |
| 20 | 34117_A GTA0189050 | {AA} | {AA} | {TT} | TGCAGGACAAGTCTTTTCCCAATCATCAGAACTCATTTTCTT[AT]ATCCAGGATAAATAAACAATTAGGAATCCTGCTATTCCAATATTGATCAA | 434 | 18 | 4.15 |
| 21 | 58876_A GTA0189053 | {GG} | {GG} | {AA} | TGCAGCAGTGTTTTCCATTTGCAGTGGTGTTCTCAATGAATATTTAGGAAGAAA[AG]CTTACAATTATAATTGGGAGTTTTGTGTTCACAGCTGG | 433 | 11 | 2.54 |
| 22 | 109335_A GTA0189077 | {TT} | {TT} | {GG} | TGCAGCTGCTGAGGGGACTGAACAACAGAGGTCAAGGGTGATGCAGATGTTGTTGGGATA[GT]TCGAGGTAGTCGGGTCAAGGGTCAAATTCTTA | 435 | 20 | 4.60 |
| 23 | 158786_B GTA0189054 | {AA} | {AA} | {GG} | TGCAGAACTAATGTCTGACCAAGTCC[AG]ACCGTCTGCTAGTAATTCCCCTCCATCAAGAGATCATAGATTATTATCTCCAATGAATAGATCAAG | 435 | 10 | 2.30 |
| 24 | 188312_A GTA0189046 | {AA} | {AA} | {TT} | TGCAGCCAACTTGTGTCCATTT[AT]ATTCTTTGCACATTTAGAATATGATCAATTGACTGTGAAGACCTTTTAAGAAATACCATTCTCATCTGTA | 433 | 9 | 2.08 |
| 25 | 61387_A GTA0189065 | {GG} | {GG} | {AA} | TGCAGTTCTCACCGACCGGTCGGATATACTAAATATTGAACG[AG]TACTGTTATTTGTTGACAATTATTTAGTGACAACAAAACATTCAATAGAC | 435 | 19 | 4.37 |
| 26 | 116758_B GTA0189079 | {AA} | {AA} | {GG} | TGCAGGAAACTGCATCAACTGATTC[AG]ATTACCATTCGCTACATGTCAATTTTCATGCATACAAAAACAAGATAAAGAAATGCATTAGATGAGA | 435 | 16 | 3.68 |
| 27 | 138581_A GTA0189080 | {AA} | {AA} | {CC} | TGCAGCATGGAATTGCATAAGAATTTGACCCTTGACCCGACTACCTCGA[AC]TATCCCAACAACATCTGCATCACCCTTGACCTCTGTTGTTCAG | 435 | 19 | 4.37 |
| 28 | 93136_A GTA0189104 | {TT} | {TT} | {CC} | TGCAGTTTTCTGTCTGTATGGTGCATTGTCTG[CT]GTGACAATTGACAACTATATTCGAATGTGTTATCCATCCATAGTTAATTTGTATTGTAAG | 434 | 19 | 4.38 |
| 29 | 161905_A GTA0189048 | {AA} | {AA} | {AG} | TGCAGACAAAGAAAATATGGATGTTTCAGATGA[AG]TCAACTAGTTTATCAGATATGAACAACAGTGCTATTGACCCAGTTACTCCTGGTAAGGG | 435 | 13 | 2.99 |
| 30 | 202081_A GTA0189103 | {TT} | {TT} | {CC} | TGCAGCAAGTCCACAGAGTTCTATTGA[CT]GAGTCCCAGGTTTTATCTCCAGAACAGGAAGCTAGTATCCCAGTGACAGTCTTGGATGAAATTGG | 435 | 19 | 4.37 |
| 31 | 239964_A GTA0189105 | {TT} | {TT} | {CC} | TGCAGGGCGCACATGTCGTTCTATTTCTATTCATCGTCTGCATGTAA[CT]AGATTTTTCCATGTGGAATTCATTTACCTGTCGAAGTCATTAATT | 434 | 17 | 3.92 |
| 32 | 37630_A GTA0189089 | {CC} | {CC} | {TT} | TGCAGAGAAACTTGATCCTTTCTTCTGTAAAGGTTGTAATAACCTTGTACA[CT]AAACCATCAGCATCCTTTGTATAATGCTGAAAGATACAATT | 434 | 17 | 3.92 |
| 33 | 7646_A GTA0189076 | {GG} | {GG} | {AA} | TGCAGAACTTAAAAAGATTCTGGAATGCAGAGA[AG]GGAAAAGTAAATGTGAATTCATTTGACACTGAGGGACAAACGGCTTTGCATCAAAGTTG | 435 | 22 | 5.06 |
